# A Local and Abscopal Effect Observed with Liposomal Encapsulation of Intratumorally Injected Oncolytic Adenoviral Therapy

**DOI:** 10.3390/cancers15123157

**Published:** 2023-06-12

**Authors:** Tao Dong, Jaimin R. Shah, Abraham T. Phung, Christopher Larson, Ana B. Sanchez, Omonigho Aisagbonhi, Sarah L. Blair, Bryan Oronsky, William C. Trogler, Tony Reid, Andrew C. Kummel

**Affiliations:** 1 Moores Cancer Center, University of California San Diego, La Jolla, CA 92037, USA; 2 Department of Chemistry and Biochemistry, University of California San Diego, La Jolla, CA 92093, USA; 3 Department of NanoEngineering, University of California San Diego, La Jolla, CA 92093, USA; 4 Materials Science and Engineering, University of California San Diego, La Jolla, CA 92093, USA; 5 EpicentRx, Inc., La Jolla, CA 92037, USA; 6 Department of Pathology, University of California San Diego, La Jolla, CA 92037, USA; 7 Department of Surgery, University of California San Diego, La Jolla, CA 92037, USA

**Keywords:** adenovirus, coxsackie and adenovirus receptor, cancer immunotherapy, TAV255

## Abstract

**Simple Summary:**

Oncolytic adenoviruses are genetically engineered to selectively replicate within cancer cells by exploiting their unique characteristics while sparing normal cells. However, adenoviruses generally require the coxsackie and adenovirus receptor (CAR) to enter the cells. This study investigated the activity of an oncolytic adenovirus in tumors with low CAR expression. The results showed that 58% and 33% of the tumors treated with liposome-encapsulated virus and viruses without liposome encapsulation achieved complete remission, respectively. Microscopic tissue analysis shows that an increased inflammatory infiltrate inside the tumor microenvironment is key to achieving effective therapeutic results in tumors independent of CAR expression. In the bilateral tumor model, both unencapsulated and encapsulated viruses reduced local tumor growth. However, only encapsulated virus reduced growth in distant tumors. Encapsulated adenovirus demonstrated an increased inflammatory infiltrate, including CD-8 and NK cells in both the treated and untreated tumors, whereas unencapsulated virus demonstrated limited inflammatory infiltrate in both tumors.

**Abstract:**

This study evaluated the in vivo therapeutic efficacy of oncolytic serotype 5 adenovirus TAV255 in CAR-deficient tumors. In vitro experiments were performed with cell lines that expressed different levels of CAR (HEK293, A549, CT26, 4T1, and MCF-7). Low CAR cells, such as CT26, were poorly transduced by Ad in vitro unless the adenovirus was encapsulated in liposomes. However, the CT26 tumor in an immune-competent mouse model responded to the unencapsulated TAV255; 33% of the tumors were induced into complete remission, and mice with complete remission rejected the rechallenge with cancer cell injection. Encapsulation of TAV255 improves its therapeutic efficacy by transducing more CT26 cells, as expected from in vitro results. In a bilateral tumor model, nonencapsulated TAV255 reduced the growth rate of the locally treated tumors but had no effect on the growth rate of the distant tumor site. Conversely, encapsulated TAV255-infected CT26 induced a delayed growth rate of both the primary injected tumor and the distant tumor, consistent with a robust immune response. In vivo, intratumorally injected unencapsulated adenoviruses infect CAR-negative cells with only limited efficiency. However, unencapsulated adenoviruses robustly inhibit the growth of CAR-deficient tumors, an effect that constitutes an ‘in situ vaccination’ by stimulating cytotoxic T cells.

## 1. Introduction

The most important achievement of clinical immunotherapy is the introduction of immune checkpoint inhibitors, which have yielded notable successes in melanoma and non-small cell lung cancer (NSCLC). In these tumor types, mutation-derived neoantigens, non-self-antigens expressed only on tumor cells, function as targets for endogenous T cells [1]. Since 2011, many immune checkpoint inhibitors (ICIs) such as anti-CTLA-4 and anti-PD1 or anti-PDL1 have been clinically employed and shown to prolong the survival of patients [2,3,4]. However, only a subset of patients responds to the ICIs due to the presence of a “cold” or immunosuppressive tumor microenvironment (TME) [5,6,7]. Several factors are associated with an immunosuppressive TME. These include the lack of tumor antigens, insufficient recruitment of antigen presentation cells (APCs), absence of T-cell priming/activations, and reduction of T-cell trafficking or infiltration to tumors [5,8,9]. Therefore, there is an unmet need to improve the clinical benefit of ICIs and other immunotherapies with modalities and strategies that convert “cold tumors” to “hot” ones.

Oncolytic viruses (OVs) are well suited to transform the TME of non-responding patients into “hot” tumors through the immunogenic liberation of tumor antigens and inflammation in situ via selective cell lysis and through the expression of therapeutic transgenes, which are carried by these “armed” OVs [10,11,12,13]. Adenoviruses are nonenveloped, icosahedral double-stranded DNA viruses that have been developed for transgene delivery in gene therapy applications and as oncolytic anticancer agents. In the case of the latter, in humans, the oncolytic adenovirus (OAd) selectively transduces tumor cells and uses them, prior to cell lysis, as processing factories for the production not only of mature virions but also for their immunostimulatory transgenes [8,14,15]. Another compelling facet of cancer immunotherapy that is gaining prominence is the combination of OAds with epigenetic drugs [16]. Epigenetic modulators have shown great potential in sensitizing resistant tumor cells to the antitumor effects of OAds, offering a promising strategy to further enhance the therapeutic efficacy of these viruses.

TAV255 is an OAd based on the human adenovirus serotype 5 (Ad5). It contains a 50 nucleotide deletion in the promoter enhancer of E1A, which restricts replication to tumor cells in humans [17]. After cell lysis, the release of tumor-associated antigens (TAAs), damage-associated molecular patterns (DAMPs), and pathogen-associated molecular patterns (PAMPs) have the potential to turn immunogenically “cold” or non-inflamed tumors into “hot” inflamed ones and, thereby, to prime antitumor adaptive immune responses against local and distant tumor sites [11,18].

The ability of OAds to transduce tumor cells selectively and to induce systemic anticancer immunity with minimal toxicity to non-malignant tissues makes them well-suited for use not only as primary therapy but also in combination with chemotherapy, targeted pathway inhibition, other immunotherapies, radiation, and surgical resection as presurgical neoadjuvant and post-surgical adjuvant therapy [19,20,21]. Despite non-chimeric OVAs being a potentially powerful tool for cancer treatment, a potential obstacle to their infection of cancer cells is low expression of coxsackie and adenovirus receptor (CAR) on some of the tumor cells [22,23,24,25]. This is because the interaction between CAR and the fiber knob protein of the viral particle dominates the internalization of Ad5 [26,27]. Most Ad serotypes enter and transduce the cells via CAR [28]. Depending on the serotype, type 5 adenovirus is the most commonly used. Ad transduction efficacy in cancer cells correlates with CAR expression, which is often downregulated in advanced cancers [29,30,31]. Over time, researchers have developed numerous modified and chimeric retargeted Ad vectors to overcome the limitation posed by CAR deficiency [31]. Such vectors as Ad5.F35, Ad5.F3 Ad5.F/RGD offer retargeting strategies that have broadened the scope of Ad application in cancer therapeutics by enabling more efficient targeting and transduction of specific tumor cells with reduced CAR expression [30,31,32]. However, a key limitation that these chimeric vectors still face is the specificity of the modified fiber knob receptor to which they are targeted. Therefore, an ultimate solution has been proposed that encapsulates the virus to enter the cells through membrane fusion, overcoming receptor limitations. In this study, a well-studied CAR-dependent oncolytic virus will be employed to focus on how CAR-dependent Ad transduces CAR-deficient tumors and how it generates immune responses.

The most common low CAR cancer cell lines, including CT26, 4T1, and MCF7, were widely used in establishing tumor models for cancer immunotherapy [32,33,34]. However, these cell lines have not yet been extensively studied in vivo for adenoviral therapy due to poor infectivity in vitro [35,36,37,38]. To overcome this issue of poor infectivity, the liposome-encapsulated adenovirus platform was developed to transduce low CAR cells efficiently in vitro [24,39,40]. A cationic liposome containing 1,2-dioleoyl-3-trimethylammonium-propane (DOTAP), cholesterol, 1,2-distearoyl-sn-glycero-3-phosphoethanolamine-N-[carboxy(polyethylene glycol)-2000] (PEG(2000)-PE carboxylic acid), 1,2-distearoyl-snglycero-3-phosphoethanolamine-N-[folate(polyethyleneglycol)-2000] (PEG(2000)-folatePE) was used recently to encapsulate the adenovirus serotype-5 including nonreplicating Ad vector expressing green fluorescent protein (GFPAd-Df) and replicating oncolytic viral vector TAV255 (TAV255-Df). Both unencapsulated GFPAd and encapsulated GFPAd-Df were tested in low CAR cells in vitro (CT26, 4T1, and MCF7).

In a previous study using a small number of mice, DOTAP-Folate liposomes encapsulated TAV255, produced by the sonication technique, efficiently treated CT26 tumors and significantly increased the population of tumor-infiltrating cytotoxic T cells. In contrast, TAV255 without encapsulation induced remission in a small fraction of the mice but showed no significant increase of T cell infiltration in a single tumor model [40]. It is noted that the TAV255 cannot replicate in mouse cancer cells either in vitro or in vivo; therefore, demonstration of remission in a mouse tumor model is challenging since multiple injections are required to mimic the natural replication to kill the cells and is consistent with tumor remission being primarily an immune response. It is noted that although human adenoviruses cannot reproduce efficiently in mouse cells, they can selectively induce dose-dependent cytotoxicity in mouse cancer cells [41]; therefore, testing in mouse models has proven valuable.

To better understand the therapeutic effects of TAV255 and TAV255-Df on the CT26 tumor model, a large group of mice was treated with higher doses in this study. In addition, extruded liposomes were used instead of sonicated liposomes for encapsulation since the former method is more practical for pharmaceutical manufacture. The therapeutic activity was observed not only with extruded encapsulated TAV255-Df but also with nonencapsulated TAV255. Based on the immunohistochemistry (IHC) of virus-transduced CT26 cells, GFP expression was observed on GFPAd and GFPAd-Df transduced cells. It is hypothesized that even a very low level of TAV255 infection in CAR-deficient tumor cells is sufficient to recruit effector cells, i.e., cytotoxic T cells and natural killer cells (NK cells) at the local injection site for the elimination of malignant cells. However, the higher transduction efficiency of TAV255-Df enhances the therapeutic antitumor effect compared to nonencapsulated TAV255.

To further study the potential for an abscopal immune response triggered by TAV255 infections whereby viral injection at one site may lead to regression of lesions at non-injected, distant sites through systemic activation of the immune system, a bilateral tumor model was used; this is the first report of therapeutic effects of encapsulated adenovirus serotype5 in a bilateral CAR-deficient tumor model. In the bilateral tumor model, unencapsulated TAV255 suppressed the growth rate of the locally treated tumor but did not mediate tumor regression at the distant uninjected site. Conversely, the encapsulated TAV suppressed the tumor growth rate not only in the directly injected lesion but also in the noninjected one. Two scenarios are possible, neither of which are mutually exclusive. (1) whereas adaptive immune responses to adenoviral coat proteins may rapidly inactivate any injected TAV255 virus that reaches the circulation, encapsulated TAV255 is likely to be more resistant to antibody neutralization and thus may “seed” the uninjected tumor site target and draw in an immune response and (2) because encapsulated virus more efficiently infects the injected tumor, it may stimulate systemic antitumor immunity more effectively in the absence of viral seeding of the uninjected tumor which the observation of greater cytotoxic T cell infiltration supports.

## 2. Materials and Methods

### 2.1. Reagents and Cell Lines

Human adenoviral vector serotype 5 expressing green fluorescence protein (GFPAd) was purchased from the Baylor College of Medicine (Vector (Burlingame, CA, USA): Ad5-CMV-eGFP 5 × 10^12^ VP/mL; 6.5 × 10^10^ PFU/mL). The CT26 (mouse colon cancer cells), 4T1 (mouse breast cancer cells), and HEK293 (human embryonic kidney cells) cell lines were purchased from American Type Culture Collection (ATCC, Manassas, VA, USA). Replicating oncolytic viral vector TAV255 (1.7 × 10^12^ VP/mL; 1.1 × 10^11^ PFU/mL), A549 (human lung cancer cells), and MCF7 (human breast cancer cells) cell lines were generously provided from the laboratory of Dr. Tony Reid. Dulbecco’s modified Eagle’s medium (DMEM) with high glucose (HyClone #SH30081.01) was supplemented with 10% of fetal bovine serum (FBS, Corning (Corning, NY, USA) #35-011-CV) and 1% of Pen Strep Glutamine (PSG, Life Technologies (Carlsbad, CA, USA) #10378-016) to prepare the complete media for HEK293, A549, and MCF7 cell culturing. Rosewell Park Memorial Institute (RPMI) 1640 (Gibco (Waltham, MA, USA) #11875093) was supplemented with 10% FBS and 1%PSG to prepare the complete RPMI for CT26 and 4T1 cell culturing. All cells were cultured at 37 °C with 5% CO_2_ in the complete media. The infectious titer assays of GFPAd and TAV255 were performed on HEK293 cells.

### 2.2. Liposome-Encapsulated Ad Synthesis

Liposome-encapsulated GFPAd and AdATP synthesis were modified based on the previous method [32,33]. DOTAP (Avanti #890890C (Glendale, AZ, USA)), cholesterol (Sigma #C3045 ((St. Louis, MO, USA))), 1,2-distearoyl-sn-glycero-3-phosphoethanolamine-N-[carboxy(polyethyleneglycol)-2000] [PEG(2000)-PE carboxylic acid, Avanti #880135P], and 1,2- distearoyl-sn-glycero-3-phosphoethanolamine-N-[folate(polyethyleneglycol)-2000] [PEG(2000)-folate-PE, Avanti #880124P] were suspended in chloroform (Sigma Aldrich Catalog #C2432) at molar ratio 1:0.26:0.02:0.01. The lipid mixture was vortexed in an amber vial for 30 min at ambient temperature (25 °C). The mixture was vacuumed overnight to form a dry lipid film. The next day, dried lipid film was hydrated with 400 µL of phosphate buffer saline pH 7.4 (PBS) (Fisher Scientific Catalog #10010072 (Waltham, MA, USA)) while vortexing. The hydrated film was stirred at 600 rpm at 4 °C for 30 min. The empty liposomes were formed by extruding the lipid solution with Avanti Mini extruder (Avanti Catalog #610000-1EA) through a 200 nm or 800 nm membrane (Cytiva/Whatman Catalog#10417004) 8 times at room temperature. The encapsulated GFPAd-Df or TAV255-Df formed by incubating the empty liposomes (Df) with GFPAd or TAV255 for 30 min at ambient room temperature. The Ad to DOTAP lipid ratio (viral particles (VP): nmol) was 5.17 × 10^7^. The unit of viral particles (VP) was used for making liposomes encapsulated Ad since the lipid-to-particle ratio was critical for transduction efficiency. PFU was used in both in vitro and in vivo transduction studies. Freshly prepared liposomes encapsulated adenovirus were used for each experiment.

### 2.3. Transduction

Cells were plated overnight at 3 × 10^4^ cells per well in a 96-well plate at 37 °C and 5% CO_2_ in the complete media. Empty liposomes Df, GFPAd, and GFPAd-Df were added to cells (day 1) at an MOI ranging from 6.3 to 100 and incubated at 37 °C with 5% CO_2_. Green fluorescence intensities were read by Tecan reader on days 2, 3, 4, 5, and 6 for HEK293, A549, MCF7, 4T1, and CT26, respectively.

### 2.4. Immunohistochemistry of Anti-GFP Staining

Tumors were harvested from the CT26-bearing BALB/c mice that received four intratumoral doses of TAV255 on days 0, 2, 4, and 6. Harvested subcutaneous tumors were trimmed to remove fat and skin before being fixed in 10% neutralized formalin for 24 h. Fixed tissues were embedded in paraffin blocks. Tissue sections were deparaffinized and rehydrated through successive alcohols (3 × xylene, 2 × 100% EtOH, 2 × 95% EtOH, 2 × 70% EtOH, deionized H_2_O). The samples were incubated in unmasking antigen solution (citrate based, pH = 6) (Vector, H-3300) at 95 °C for 30 min for antigen retrieval. Samples were incubated with peroxidase block Bloxall (Vector, SP-6000) for 10 min, followed by protein block Blotto (Thermo, PI37530) for 10 min. Afterward, the samples were incubated with anti-GFP primary antibody (Rabbit, Cell Signaling, 2555S, 1:2000) for 1 hr and followed by anti-rabbit HRP polymer (Cell IDX, 2RH-100, RTU) for 30 min. DAB (brown) Chromogen (VWR, 95041-478) and Mayer’s hematoxylin (Sigma, 51275) were used to stain the sample. The number of cells expressing GFP, which is indicative of Ad transduced cells, was determined by counting positive cells in twelve different randomly selected fields. All sections were analyzed under a brightfield microscope.

### 2.5. Cell Viability Assay

Cells were seeded in 96-well plates at a density of 3 × 10^4^ cells/well 24 h before viral infection. Cells were infected with TAV255 at an MOI of 0, 1, 10, 100, 1000, and 10,000 for 48 h (n = 3). Cell viability was determined using the alamarBlue™ HS Cell Viability Reagent (Invitrogen, Eugene, OR, USA) according to the manufacturer’s protocol.

### 2.6. Animal Studies

Six- to eight-week-old female BALB/cAnNHsd mice were purchased from Envigo RMS, LLC. Mice were housed in high-efficiency particulate air (HEPA) cages in a specific pathogen-free facility with food and water available and a 12 h light/dark cycle. 1 × 10^6^ CT26 cells in 50 μL of PBS were subcutaneously injected on the right side of the flank or both sides of the flanks to establish the single or bilateral tumor model, respectively. Tumor volume was determined by a caliper with the modified ellipsoidal formula: volume (mm^3^) = (width × width × length)/2, and treatment was started at a tumor size that reached 20 mm^3^. 3.2 × 10^8^ PFU of TAV255 or TAV255-Df, or equivalent Df, or PBS was intratumorally injected every other day. Pain and distress in tumor-bearing mice were closely monitored. For the survival study, mice were euthanized when a tumor ulcerated or reached 1500 mm^3^. All procedures and protocols were approved by the UC San Diego Institutional Animal Care and Use Committee (IACUC).

### 2.7. Multiplex Immunofluorescence Staining

Biliteral tumors were harvested from both sides of the CT26 bearing BALB/c mice that received four intratumoral doses of PBS, TAV255 and TAV255-Df in their primary tumors on days 0, 2, 4, and 6. Harvested tumors were fixed and paraffin-embedded before sectioning. Sectioned samples were incubated in antigen unmasking solution (citrate based, pH = 6) (Vector, H-3300) at 95 °C for 30 min for antigen retrieval. Samples were incubated with peroxidase block Bloxall (Vector, SP-6000) for 10 min, followed by mouse IgG block for 1 hr and protein block Blotto (Thermo, PI37530) for 10 min, respectively. Afterward, the samples were incubated with primary antibodies, including anti-CD8 (Rat, Invitrogen, 14-0195-82, 1:50) and anti-NK1.1 (Mouse, Thermo, MA1-70100, 1:100) for 1 hr. Secondary antibodies were incubated for 30 min before mounting. The number of cells expressing CD8 and NK1.1, which is indicative of CD8+ T cells and NK cells, was determined by counting positive cells in three to six different randomly selected fields. All sections were analyzed under a fluorescence microscope.

### 2.8. RT-qPCR Analysis

Total RNA was extracted from cells using the PureLink™ RNA Mini Kit (Invitrogen™, Carlsbad, CA, USA) after tissue digestion. Reverse transcription reactions were performed at 25 °C for 10 min, 37 °C for 120 min, 85 °C for 5 min, and 4 °C for 5 min by cDNA Reverse Transcription Kit (Applied Biosystems, Waltham, MA, USA). PCR amplification was conducted with 40 cycles of denaturation at 95 °C for 20 s and annealing at 60 °C for 20 s. The threshold cycle values for E1A were obtained using a ViiA 7 real-time qPCR system (Life Technologies, Carlsbad, CA, USA) and converted to the gene copy number from the standard curves. Primers and probes were synthesized by the manufacturer (Integrated DNA Technologies, San Diego, CA, USA): E1A probe, 5′-AGCCCGAGCCAGAACCGGAGCCTGCAA-3′; E1A primers, forward, 5′-TGTGTCTGAACCTGAGCCTG-3′, reverse, 5′-ATAGCAGGCGCCATTTTAGG-3′.

### 2.9. Statistical Analysis

Prism 9 software (GraphPad Software, La Jolla, CA, USA) was used for data analysis. Comparison between two groups was based on a two-tailed unpaired t-test. Log-rank test was performed to establish the significance of the differences between survival data. A value of *p* < 0.05 was determined to be statistically significant.

## 3. Results and Discussion

### 3.1. Transduction of CAR^+^ and CAR^−^ Cells by Ad with or without Encapsulation In Vitro

**Figure 1:**
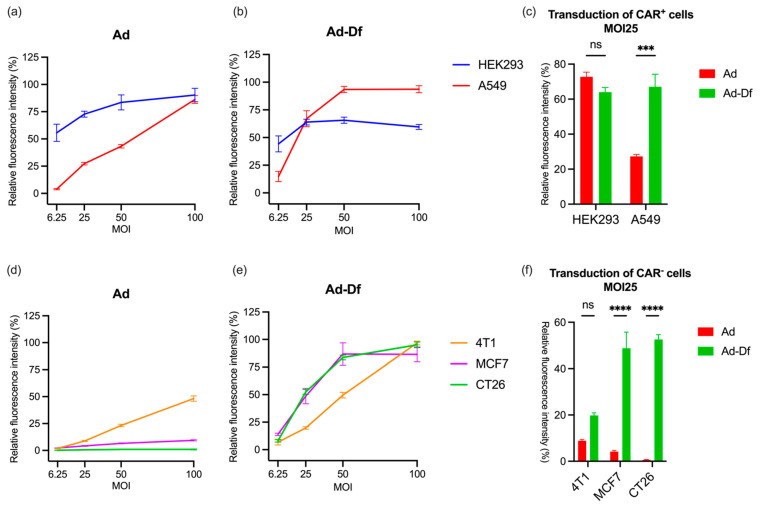
**Transduction of various cell lines with Ad and Ad-Df.** Transduction of high CAR level (CAR^+^) cells HEK293 and A549 with unencapsulated adenovirus (Ad) (**a**), and liposome-encapsulated adenovirus (Ad-Df) (**b**) at MOI 6.25, 25, 50, and 100. GFP expression on CAR^+^ cells by transduction of Ad and Ad-Df at MOI 25 (**c**). Transduction of low CAR (CAR^−^) cells 4T1, MCF7, and CT26 with Ad (**d**) and Ad-Df (**e**) at MOI 6.25, 25, 50, and 100. GFP expression on CAR- cells by transduction of Ad and Ad-Df at MOI 25 (**f**). Fluorescence intensities were read on days 2, 3, 4, 5, and 6 for HEK293, A549, MCF7, 4T1, and CT26, respectively. Data are shown as mean (n = 3) with standard errors of the mean (SEM). ns = no significant; *** *p* < 0.001, **** *p* < 0.0001.

Replication-deficient green fluorescent protein (GFP)-expressing Ad vectors (GFPAd) were used to evaluate the infection ability by quantifying the fluorescence level of infected cells. HEK293 (human embryonic kidney cells), A549 (human lung cancer cells), CT26 (mouse colon cancer cells), MCF-7 (human breast cancer cells), and 4T1 (mouse breast cancer cells) were used to determine the infectivity of the unencapsulated Ad and extruded liposome encapsulated adenovirus (Ad-Df) with varying CAR expression. The CAR expression levels on the cells were reported previously and affected the transduction rate [41]. HEK293 and A549 cells expressed more CAR than 4T1, MCF7, and CT26 cell lines, which are usually considered to be low CAR-expressing (CAR-) cells due to the downregulation of the receptor (Table S1). Diluted unencapsulated Ad and DOTAP liposomes encapsulated Ad-Df with PBS were added to the cells in a 96-well plate at MOIs 6.25, 25, 50, and 100. Due to the different transduction rates related to CAR expression levels, 2–6 days of incubation of Ad/Ad-Df with cells were performed. The plates were read with a fluorometer, and fluorescence was normalized based on cells with PBS only. Infection of HEK293 and A549 cells was very efficient due to the high level of the CAR expression among these cell lines (Figure 1a). Even in a high CAR-expressing (CAR+) cell line, higher GFP expression was observed in Ad-Df-transduced A549 compared to unencapsulated Ad-transduced cells (Figure 1b,c). The fluorescence signal on Ad-Df infected HEK293 drops at high MOIs due to the cytotoxicity of the Ad-Df at high concentrations. These results suggest that both Ad and Ad-Df effectively transduce CAR^+^ cells.

Moderate to low GFP expression was observed on Ad-transduced 4T1, MCF7, and CT26 cells (Figure 1d). Unencapsulated Ad did not effectively transduce these cells due to CAR levels below detection levels on the surface of MCF7 and CT26. In contrast, the encapsulated Ad-Df infected all three CAR^−^ cell lines effectively with similar GFP expression, which suggests that the infection of Ad-Df is independent of CAR levels (Figure 1e). At MOI 25, the fluorescence intensity of Ad-Df transduced A549, MCF7, and CT26 was 3-, 12-, and 88-fold higher than that of unencapsulated Ad transfected cells, respectively (Figure 1c,f). The results suggested that the transduction of CT26, MCF7, and A549 by Ad-Df was significantly improved compared with unencapsulated Ad. Empty liposomal Df was used as a control, and no fluorescence was observed in all five cell lines (Figure S1). These results indicate that low CAR-expressing MCF7 and CT26 cells are associated with poor transduction efficiency in the absence of liposomal encapsulation. Encapsulation enhances the transduction of 4T1 cells, albeit not as effectively as in the cases of CAR-deficient MCF7 and CT26 cells. This disparity might be attributed to different expression levels of folate receptors (FR) among these cell lines. Literature suggests that FR is overexpressed in both CT26 and MCF-7 cells [42,43], whereas 4T1 cells, commonly used as a model for FR-negative cells, demonstrate considerably lower FR expression [44].

### 3.2. Ad Transduces CT26 Cells at High MOI as Confirmed by High Sensitivity Immunohistology Staining

**Figure 2:**
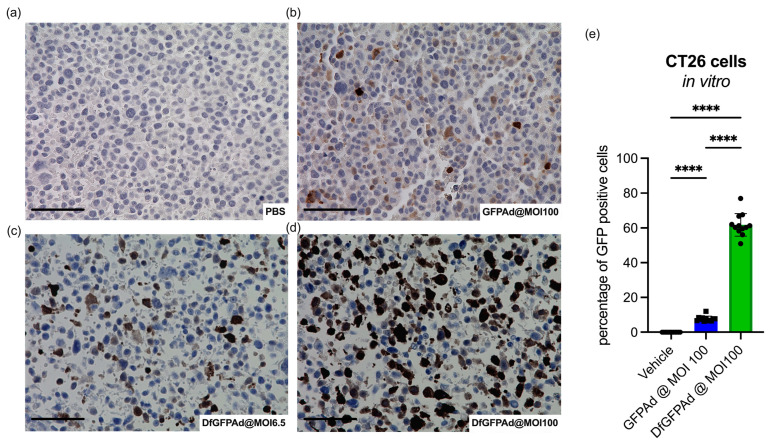
**Transduction of CT26 and cell viability with unencapsulated virus.** IHC slides show anti-GFP staining of GFPAd transduced CT26 cells. A comparison between the control PBS (**a**), unencapsulated GFPAd at MOI 100 (**b**), liposome-encapsulated GFPAd-Df at MOI 6.5 (**c**), and GFPAd-Df at MOI 100 (**d**), transduced CT26 cells. Quantification of stained GFP-positive cells based on the IHC slides (**e**). Mean GFP positive cells based on IHC images of n = 12 and standard deviations are shown. scale bar = 50 μm, **** *p* < 0.0001.

GFP is a reporter protein that can be easily detected by fluorescence. However, the sensitivity may be limited due to the weak fluorescence signals that are unevenly distributed in samples [45]. When Ad infects only a small number of cells, faded or weak GFP signals may result in GFPAd transduction that is hard to detect. To better understand the infectivity of Ad with CAR^−^ CT26 cells, anti-GFP staining was used on fixed cells with unencapsulated Ad and Ad-Df transduction. CT26 cells were plated on 6-well plates and incubated with GFPAd and GFPAd-Df at different MOIs. PBS was added to the cells as a control for the experiment. After 4 days of incubation, cells were fixed and stained with anti-GFP antibodies. A small amount of CT26 cells infected with unencapsulated GFPAd was stained brown compared to the control cells (Figure 2a,b). In contrast, GFPAd-Df transduced CT26 cells very efficiently at MOI 6.5 (Figure 2c) and MOI 100 (Figure 2d), as shown by more extensive and stronger brown staining. These results indicate that the transduction of unencapsulated GFPAd in CT26 cells is very low but detectable. Although the fluorescence intensity is close to zero in Figure 1, GFPAd, in fact, transduces about 7.8% ± 1.5 of the cells, as the quantification of many images demonstrates. Conversely, 8X more GFPAd-Df transducted cells were GFP positive (61.8% ± 6.5) compared to unencapsulated GFPAd transduced cells. GFP staining more accurately assessed that unencapsulated Ad transduced CT26 in small amounts at high MOI, while Ad-Df effectively transduced CT26 at both low and high MOIs, which is consistent with Figure 1. The fact that GFPAd and GFPAdDf transduce CT26 at different levels makes CT26 a feasible and relevant syngeneic tumor model to study the therapeutic efficacy of encapsulated and nonencapsulated Ad.

### 3.3. Anti-Tumor Effects of Unencapsulated and Encapsulated TAV-255 in a CT26 Subcutaneous Tumor Model

Accordingly, the potential therapeutic effects of the Ad-Df on a CAR-deficient tumor were investigated in vivo in a CT26 murine model. Murine cells do not support adenoviral replication [15]; therefore, any effects observed in this animal model are secondary to immune activity rather than oncolysis, as would be observed in humans. The experiment was designed based on a previous study [40]. The BALB/c mice were subcutaneously inoculated with 1 × 10^6^ CT26 cells in the right flank. Tumor volume was monitored daily until it reached 50 mm^3^ to start treatment. Four treatment groups were used to evaluate the therapeutic effect of TAV255 on CAR-deficient tumors: vehicle group injected PBS (n = 10), extruded empty liposomes (Df) (n = 10), unencapsulated TAV255 (n = 24), and extruded liposome-encapsulated TAV255 (TAV255-Df) (n = 24). Extruded liposomes were used instead of sonicated liposomes for encapsulation since the former is a manufacturable method, and no significant therapeutic difference was observed in an animal study (Figure S2). The agents were intratumorally injected every other day for twenty doses to mimic the effects of a replicating virus in a human tumor model. Each dose contained 8.2 × 10^8^ pfu in 100 uL solution. Survival data were analyzed using the log-rank test; all mice in the control group, including PBS and Df, reached the endpoint before completing the treatments.

**Figure 3:**
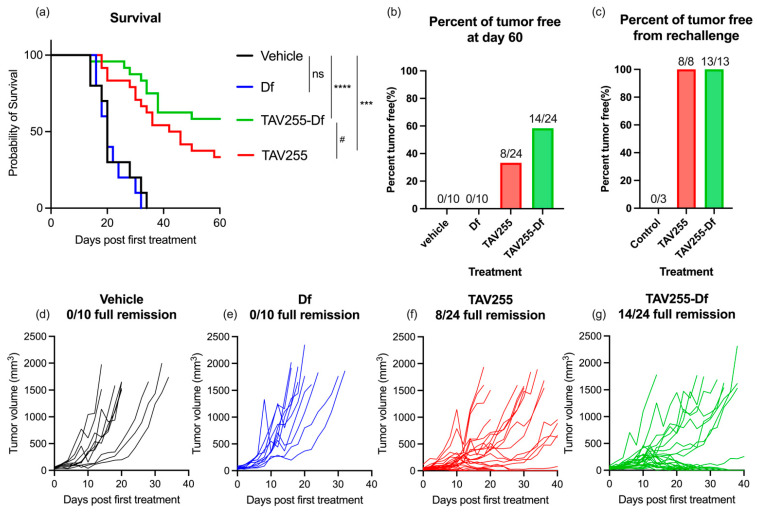
**Comparison of unencapsulated vs. encapsulated TAV255 on CAR-deficient tumor growth and remission**. Treatment schedule: PBS, empty liposome (Df), TAV255, and TAV255-Df (n = 10–24) were intratumorally injected every other day. Survivors with full remission were rechallenged by CT26 tumors on day 64. (**a**) Survival curves (vehicle n = 10, empty liposomes n = 10, unencapsulated adenovirus TAV255 n = 24 and liposomes encapsulated TAV255-Df n = 24) with initial challenge of CT26 tumor on the right flank of the mice and rechallenge of the tumors on the left flank. (**b**) 33% of the mice had complete remission from the treatment of TAV255, and 58% of the mice had complete remission by TAV255-Df. (**c**) 8/8 and 13/13 mice from TAV255 and TAV255-Df treatments survived from the rechallenge. Individual tumor curves of vehicle (**d**), Df (**e**), TAV255 (**f**), TAV255-Df (**g**). *p* values compare survival curves with a log-rank (Mantel–Cox) test. ns = no significant; # *p* = 0.1, *** *p* < 0.001; **** *p* < 0.0001.

Both unencapsulated TAV255 and liposome-encapsulated TAV255-Df not only significantly delayed the tumor progression, *p* < 0.001 and *p* < 0.0001, respectively, but also eradicated some of the tumors at the end of the study (Figure 3a,f,g). No significant difference was observed between the vehicle PBS vs. empty liposome Df, suggesting that empty liposomes have no therapeutic effect (Figure 3d,e). After treatment with TAV255, 33% (8/24) of the tumors went into full remission. The result suggested that TAV255 suppressed the CT26 tumor even without encapsulation. Compared to TAV255, TAV255-Df was nearly two-fold more effective, with 58% (14/24) of tumors having achieved complete remission (Figure 3b). The mice with complete remission were monitored closely for three weeks, and none of them experienced tumor recurrence. Since in vitro unencapsulated TAV255 is not directly lytic to CT26 cells below MOI200 (Figure S3), an immune response to the TAV255-infected tumors is mainly responsible for the anti-cancer effect.

To investigate the potential immune response generated during the treatment with TAV255 and TAV255-Df, a re-challenge of CT26 tumors was injected into the opposite flank from the initial tumor site. The mice with complete remission were rechallenged by injecting 1 × 10^6^ CT26 cells in the left fanks after 24 days from the last treatment to allow them to clear the residual virus. All survivors with full remission of the initial tumors from both TAV255 and TAV255-Df groups rejected the re-challenge of CT26 (Figure 3c). The tumor-free mice were monitored for up to three months, and none of them recurred. Control naive mice were challenged with the same dose of CT26 from the re-challenge study, and all mice failed to reject CT26. CD8+ T cells have been reported in the literature to respond effectively to both tumor and adenovirus antigens [46]. These results strongly suggest that long-term CT26 tumor-specific immune responses develop from the TAV255-infected tumors. However, more detailed experiments are needed to identify whether CD8 and/or humoral memory cells are responsible for the observed therapeutic effect.

### 3.4. Unencapsulated and Encapsulated TAV255 Suppresses Tumor Growth in a CT26 Bilateral Tumor Model

**Figure 4:**
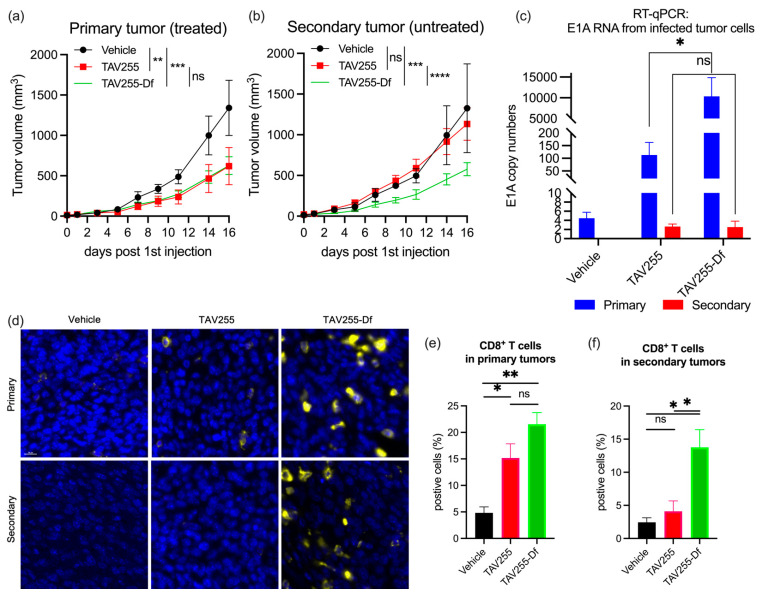
**Therapeutic effects of unencapsulated TAV255 and encapsulated TAV255-Df on a bilateral tumor model.** CAR-deficient tumors were inoculated on both sides of the BALB/c mice. The tumors that received the direct injections were denoted as “primary” tumors, and the tumor that did not receive direct injections were denoted as “secondary” tumors. (**a**) Average growth curve of the primary tumors from vehicle (n = 3), TAV255 (n = 6), and TAV255-Df (n = 6) treated mice. (**b**) Average growth curve of the secondary tumors from vehicle (n = 3), TAV255 (n = 6), and TAV255-Df (n = 6) treated mice. (**c**) E1A copy numbers from both primary and secondary tumors transduced by unencapsulated TAV255 or encapsulated TAV255-Df. (n = 6). (**d**) Immunofluorescence staining of CD8+ T cells. DAPI-stained cells shown in blue, and CD8+ cells shown in yellow. (**e**,**f**) Quantification of the CD8+ T cells in both primary and secondary tumors based on immunofluorescent staining (n = 3–6). Data are shown as mean with standard errors of the mean (SEM). scale bar = 10 μm. ns = no significant difference; * *p* value < 0.05; ** *p* value < 0.01; *** *p* value < 0.001, **** *p* value < 0.0001.

To evaluate for an abscopal effect, a bilateral CT26 tumor model in immunocompetent BALB/c mice was used in which only one of the tumors received intratumoral (i.t.) injections of either vehicle, TAV255, or TAV255-Df every other day for four doses. Unencapsulated TAV255 led to regression of the primary CT26 tumor with a *p*-value < 0.01. Encapsulated TAV255-Df led to regression of the primary tumor with a *p*-value < 0.001 (Figure 4a) consistent with previous results from the single tumor model (Figure 3). However, unlike the anenestic or abscopal effect observed with encapsulated TAV255, unencapsulated TAV255 did not induce regression in the uninjected contralateral tumor (Figure 4b).

To investigate the hypothesis that the encapsulated TAV255-Df could be “leaking” from the injected tumor site to untreated distant tumor sites, an RT-qPCR study was performed to analyze the E1A copy numbers in both primary and secondary tumors. As shown in Figure 4c, no E1A expression was observed in tumors from control mice who only received PBS. Similarly, no E1A expression was found in all secondary tumors, irrespective of whether the primary tumors received TAV255 or TAV255-Df treatments. This observation supports the hypothesis that TAV255-Df is not leaking from the treated site to a distant tumor. Unencapsulated TAV255-treated primary tumors exhibited low levels of E1A expression, consistent with the hypothesis that CAR-dependent Ads transduce CAR-negative cells in vivo with very low efficiency. However, a significant amount of E1A RNA was observed in the primary tumor cells transduced with liposome-encapsulated TAV255-Df. This observation is consistent with previous in vitro data demonstrating that encapsulated CAR-dependent Ads can efficiently transduce CAR-negative cells. Compared to unencapsulated TAV255, encapsulation amplified the E1A transcription by 92-fold, which could potentially enhance overall systemic antitumor immunity more effectively, even in the absence of viral seeding of the uninjected tumor (Figure 4c). To further validate this concept, the cytotoxic T-cell infiltration from both tumors was investigated.

To confirm that CD8+ T cells infiltrated into tumor tissues after TAV255 and TAV255-Df treatment, fixed primary and secondary tumors from treated mice were stained with anti-CD8 antibodies (Figure 4d). In a previous study, a lower dose (4.3 × 10^8^ pfu/50 uL) of intratumorally injected TAV255 did not significantly inhibit tumor growth compared to the vehicle [41]. This is possibly secondary to little or no intratumoral T-cell infiltration at this low dose. However, at this higher dose (8.2 × 10^8^ pfu 100 uL), a 3–4-fold increase in CD8+ T cells population was observed in TAV255 and TAV255-Df transduced primary tumors compared to vehicle-treated ones (Figure 4e). This indicates that even in the absence of high transduction efficacy (Figure 2), the infiltration of cytotoxic T cells is likely sufficient to convert “cold” CAR^−^ CT26 primary tumors into hot ones with immune cells that eradicate them. However, the CD8+ T cell population was not significantly increased in secondary tumors after treatment with unencapsulated TAV255 compared to the vehicle alone (Figure 4f). This result confirms the fact that the abscopal effect requires the presence of tumor-specific adaptive immunity. Nevertheless, although systemic tumor immunity did not develop immediately after TAV255 administration, it may have developed after more time. In contrast, the population of CD8+ T cells and NK cells were significantly increased in TAV255-Df-treated secondary tumors (Figure S4). This suggests two non-mutually exclusive scenarios; as previously stated, either the encapsulated virus travels to the contralateral tumor and triggers an immune response there, or local injection activates a strong and immediate systemic immune response against the uninjected tumor.

## 4. Conclusions

Low expression levels of CAR, the primary Ad receptor on tumor cells, limits the infection efficiency of CAR-dependent oncolytic Ad. Despite that chimeric adenoviral vectors have emerged as a strategy to overcome the limitation of low CAR expression on tumor cells, a key limitation that these chimeric vectors still face is the specificity of the modified fiber knob receptor to which they target. Therefore, an ultimate solution has been proposed that encapsulates the virus to enter the cells through membrane fusion, overcoming receptor limitations. This study explored the impact of CAR-dependent oncolytic adenoviruses both in vitro and in vivo, with or without its encapsulation. Even though cells with CAR expression below the limit of detection are not efficiently transduced by unencapsulated adenovirus in vitro, an unencapsulated oncolytic adenovirus, TAV255, induced complete remission in 33% of mice bearing CT26 tumor xenografts. Since unencapsulated TAV-255 is not directly lytic in these mouse cells, this suggests that through an “in situ vaccination” effect, the unencapsulated TAV255-infected cells recruit and possibly activate CTLs against the tumor even in the absence of direct lysis, which is consistent with the presence of immune cells observed in immunostained tumors. The encapsulated TAV255-Df markedly enhanced local infection and produced an abscopal effect, which slowed growth in both treated and distal tumor sites. Limitations of this study are several and include (1) a longer time may have been needed to determine whether unencapsulated TAV255 elicits an abscopal effect since treatment responses generally are slower to manifest for immune therapy than for other conventional treatments especially given the inefficiency of transduction in CT26 tumors; (2) an evaluation was not performed of the ratio of tumor-infiltrating CD8^+^T effector cells to immunosuppressive FoxP3^+^ T_regs_ and to what extent that ratio correlates with the extent of tumor shrinkage since the higher the ratio of CD8 T cells/T_regs_ usually the more favorable the outcome in many tumors [47].

TAV255 is the base vector for an armed oncolytic virus called AdAPT-001, currently in a Phase 1/2 clinical trial (NCT04673942). Based on the evidence of activity from these experiments, encapsulation of TAV255 increases immune cell infiltration into the tumor matrix and improves the pathological complete response rate by a transduction enhancement both in vitro and in vivo. It constitutes a new local and systemic therapy in the armamentarium against cancer, with the potential to render “cold”, poorly infiltrated tumors “hot“, and more therapeutically responsive and also to prevent a metastatic recurrence. Future research should further explore the potential immune-stimulatory effects of encapsulated Ads and assess their benefits when combined with other therapeutic strategies, such as checkpoint inhibitors.

## Data Availability

The data presented in this study are available in articles and supplementary material.

## Supplementary Materials

The following supporting information can be downloaded at: https://www.mdpi.com/article/10.3390/cancers15123157/s1. Table S1: Coxsackie and Adenovirus Receptor (CAR) expression levels. Figure S1. GFP signals from empty liposomes incubated cells Incubation of high CAR level (CAR+) cells HEK293 and A549 and low CAR level cells CT26, MCF7 and 4T1 with empty liposomes at the concentration equivalent to Ad-Df at MOI 6.25, 25, 50 and 100. Fluorescence intensities were read with a Tecan reader on day 2, 3, 4, 5, 6 for HEK293, A549, MCF7, 4T1, and CT26, respectively. Data are shown as mean (n = 3) with standard errors of the mean (SEM). No significance was observed between each cell line and all values are close to 0. Figure S2. Comparison of encapsulated Ad made by extrusion or sonication on CAR-deficient tumor model Survival curves (vehicle n = 5, encapsulated Ad made by extrusion n = 6, encapsulated Ad made by sonication n = 5) with initial challenge of CT26 tumor on the right flank of the mice and followed by injections every other day. *p* values compare survival curves with a log-rank (Mantel-Cox) test. ns = no significant; * *p* < 0.05. Figure S3: The viability of oncolytic adenovirus TAV255 and encapsulated TAV255-Df infected CT26 CT26 were incubated with TAV255 or TAV255-Df at MOI 13, 25, 50, 100, and 200 for 5 days. AlmarBlue assay was performed on infected cells and read fluorescence by Tecan. Data are shown as mean (n = 3) with standard errors of the mean (SEM). The killing activity by TAV255-Df occurs starting at MOI 25. Figure S4: NK cells from biliteral tumor model CAR-deficient tumors CT26 were inoculated on both sides of the BALB/c mice. The tumors that received the direct injections were denoted as “primary” tumors, and the tumor that did not receive direct injections were denoted as “secondary” tumors. Quantifications of the CD8+ T cells in both primary and secondary tumors based on immunofluorescent staining of NK1.1 (n = 3–6). Data are shown as mean with standard errors of the mean (SEM). ns = no significant difference; * *p* value < 0.05; ** *p* value < 0.01; *** *p* value < 0.001, **** *p* value < 0.0001.
