# Supplementary material for: A Local and Abscopal Effect Observed with Liposomal Encapsulation of Intratumorally Injected Oncolytic Adenoviral Therapy
Source: Cancers (Basel). 2023 Jun 12;15(12):3157. doi: 10.3390/cancers15123157 (PMC10296131; doi:10.3390/cancers15123157)
Supplement: Supplementary file 1 [file cancers-15-03157-s001.zip › cancers-2257137-supplementary.pdf]

Supplementary Material: A local and abscopal effect observed with liposomal encapsulation of intratumorally injected oncolytic adenoviral therapy

Tao Dong, Jaimin R. Shah, Abraham T. Phung, Christopher Larson, Ana B. Sanchez, Omonigho Aisagbonhi, Sarah L. Blair, Bryan Oronsky, William C. Trogler, Tony Reid, and Andrew C. Kummel*

Table S1: Coxsackie and Adenovirus Receptor (CAR) expression levels

	Name	CAR expression*
**HEK293**	human embryonic kidney cells	+++++
**A549**	human lung cancer cells	++++
**MCF7**	human breast cancer cells	+
**CT26**	mouse colon cancer cells	Ø**

*The CAR expression evaluation based on literatures [1,2]. **non-detectable CAR expression

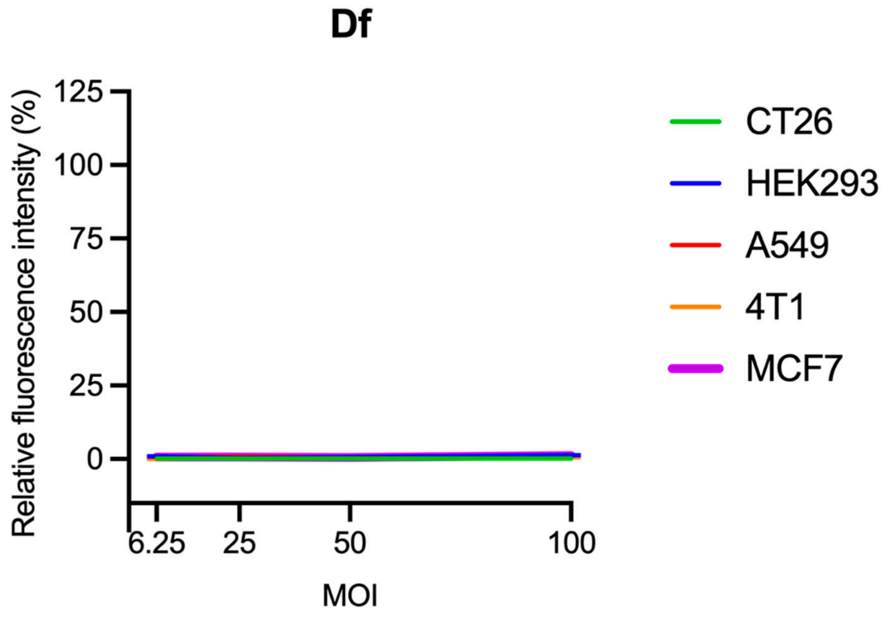

Figure S1 GFP signals from empty liposomes incubated cells Incubation of high CAR level (CAR+) cells HEK293 and A549 and low CAR level cells CT26, MCF7 and 4T1 with empty liposomes at the concentration equivalent to Ad-Df at MOI 6.25, 25, 50 and 100.

Fluorescence intensities were read with a Tecan reader on day 2, 3, 4, 5, 6 for HEK293, A549, MCF7, 4T1, and CT26, respectively. Data are shown as mean (n=3) with standard errors of the mean (SEM). No significance was observed between each cell line and all values are close to 0.

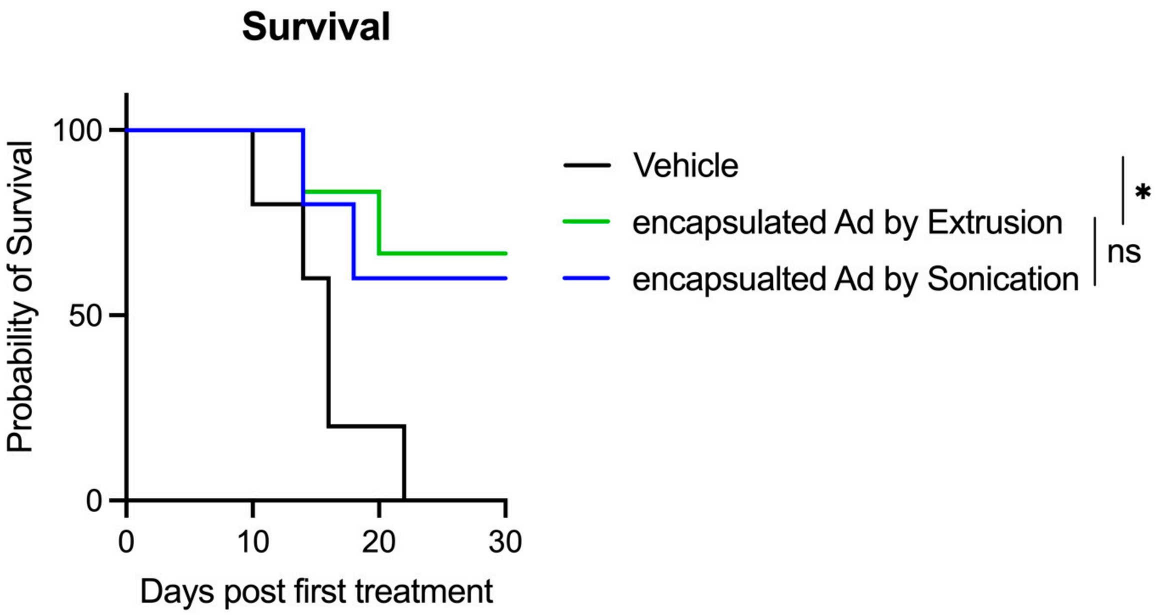

Figure S2 Comparison of encapsulated Ad made by extrusion or sonication on CAR-deficient tumor model Survival curves (vehicle n=5, encapsulated Ad made by extrusion n=6, encapsulated Ad made by sonication n=5) with initial challenge of CT26 tumor on the right flank of the mice and followed by injections every other day. p values compare survival curves with a log-rank (Mantel-Cox) test. ns = no significant; $*p < 0.05$

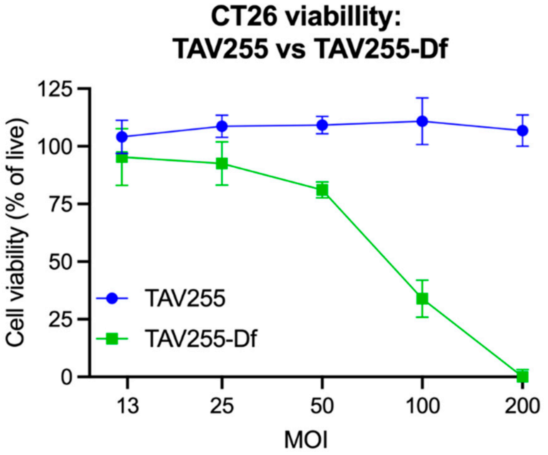

Figure S3 The viability of oncolytic adenovirus TAV255 and encapsulated TAV255-Df infected CT26 CT26 were incubated with TAV255 or TAV255-Df at MOI 13, 25, 50, 100, and 200 for 5 days. AlmarBlue assay was performed on infected cells and read fluorescence by Tecan. Data are shown as mean (n=3) with standard errors of the mean (SEM). The killing activity by TAV255-Df occurs starting at MOI 25.

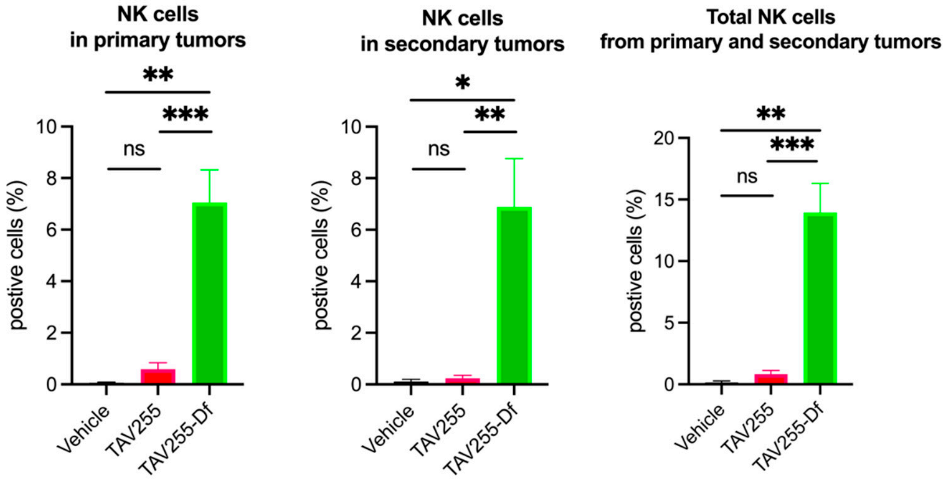

Figure S4 NK cells from biliteral tumor model CAR-deficient tumors CT26 were inoculated on both sides of the BALB/c mice. The tumors that received the direct injections were denoted as "primary" tumors, and the tumor that did not receive direct injections were denoted as "secondary" tumors. Quantifications of the CD8+ T cells in both primary and secondary tumors based on immunofluorescent staining of NK1.1 (n = 3 - 6). Data are shown as mean with standard errors of the mean (SEM). ns = no significant difference; *p value < 0.05; ** p value < 0.01; ***p value < 0.001, ****p value < 0.0001.

References

1. Huang CH, Dong T, Phung AT, Shah JR, Larson C, Sanchez AB, Blair SL, Oronsky B, Trogler WC, Reid T, Kummel AC. Full Remission of CAR-Deficient Tumors by DOTAP-Folate Liposome Encapsulation of Adenovirus. ACS Biomater Sci Eng. 2022 Nov 17. doi: 10.1021/acsbiomaterials.2c00966. Epub ahead of print. PMID: 36395425.
2. Shah, J.R.; Dong, T.; Phung, A.T.; Reid, T.; Larson, C.; Sanchez, A.B.; Oronsky, B.; Blair, S.L.; Aisagbonhi, O.; Trogler, W.C.; Kummel, A.C. Development of Adenovirus Containing Liposomes Produced by Extrusion vs. Homogenization: A Comparison for Scale-Up Purposes. Bioengineering 2022, 9, 620. https://doi.org/10.3390/bioengineering9110620
